# ANNOUNCEMENT / NOUVELLE

**DOI:** 10.29173/jchla29772

**Published:** 2024-04-01

**Authors:** Christine Neilson

## Join us in Winnipeg from June 11 to 14, 2024, for our annual conference!

There is a great program on offer this year. In addition to content contributed by health library colleagues from across Canada and abroad, we are excited to offer three keynote presenters: Catherine Wreford, 2022 winner of *The Amazing Race Canada*, Joe Curnow, “a scholar of learning, identity development, and youth-led social change,” and writer, podcaster, educator, and activist Nora Loreto. But conference isn’t all work and no play – there are opportunities to network with colleagues at the Winnipeg Art Gallery-Qaumajuq during the reception on Tuesday, during an informal dine-around dinner on Wednesday, and the banquet on Thursday. We have also arranged for local tours before and after the conference so you can experience Winnipeg. See the conference website for details.

Registration is now open, and early bird pricing is in effect until May 10, 2024. We hope to see you in Winnipeg!

**Christine Neilson**

2024 Conference Planning Committee, Publicity

## Rejoignez-nous à Winnipeg du 11 au 14 juin 2024 pour notre conférence annuelle!

Le programme de cette année est excellent. En plus du contenu apporté par les collègues des bibliothèques de santé de tout le Canada et de l'étranger, nous sommes ravis d'offrir trois conférencières invitées : Catherine Wreford, gagnante de The Amazing Race Canada en 2022, Joe Curnow, “spécialiste de l'apprentissage, du développement de l'identité et du changement social mené par les jeunes", et Nora Loreto, écrivain, podcasteur, éducatrice et activiste. Mais la conférence n'est pas que du travail - il y a des occasions de nouer des contacts avec des collègues à la Winnipeg Art Gallery-Qaumajuq pendant la réception du mardi, pendant un dîner informel le mercredi, et pendant le banquet du jeudi. Nous avons également organisé des visites locales avant et après la conférence pour vous permettre de découvrir Winnipeg. Voir le site web de la conférence pour plus de détails.

L'inscription est maintenant ouverte, et le prix pour les inscriptions anticipées est en vigueur jusqu'au 10 mai 2024. Nous espérons vous voir à Winnipeg!

**Christine Neilson**

*Comité de planification de la conférence 2024, publicité*

